# Upregulated spinal histone deacetylases induce nociceptive sensitization by inhibiting the GABA system in chronic constriction injury–induced neuropathy in rats

**DOI:** 10.1097/PR9.0000000000001209

**Published:** 2024-11-06

**Authors:** Zhi-Hong Wen, Nan-Fu Chen, Hao-Jung Cheng, Hsiao-Mei Kuo, Pei-Yu Chen, Chien-Wei Feng, Zhi-Kang Yao, Wu-Fu Chen, Chun-Sung Sung

**Affiliations:** a Department of Marine Biotechnology and Resources, National Sun Yat-sen University, Kaohsiung, Taiwan; b Institute of Biopharmaceutical Sciences, National Sun Yat-sen University, Kaohsiung, Taiwan; c Institute of Medical Science and Technology, National Sun Yat-sen University, Kaohsiung, Taiwan; d Division of Neurosurgery, Department of Surgery, Kaohsiung Armed Forces General Hospital, Kaohsiung, Taiwan; e Department of Neurosurgery, Kaohsiung Chang Gung Memorial Hospital and Chang Gung University College of Medicine, Kaohsiung, Taiwan; f Department of Obstetrics and Gynecology, Kaohsiung Medical University Hospital, Kaohsiung Medical University, Kaohsiung, Taiwan; g Department of Orthopedics, Kaohsiung Veterans General Hospital, Kaohsiung, Taiwan; h Division of Pain Management, Department of Anesthesiology, Taipei Veterans General Hospital, Taipei, Taiwan; i School of Medicine, National Yang-Ming Chiao Tung University, Taipei, Taiwan

**Keywords:** HDAC, Panobinostat, Neuropathic pain, GAD65, GABA, CCI

## Abstract

**Introduction:**

Neuropathic pain (NP) affects countless people worldwide; however, few effective treatments are currently available. Histone deacetylases (HDACs) participate in epigenetic modifications in neuropathy-induced nociceptive sensitization. Gamma-aminobutyric acid (GABA) is a major inhibitory neurotransmitter that can inhibit NP. The present study aimed to examine the role of spinal HDAC and its isoforms in neuropathy.

**Methods:**

Male Wistar Rat with chronic constriction injury (CCI)-induced peripheral neuropathy and HDAC inhibitor, panobinostat, was administrated intrathecally. We performed quantitative real-time polymerase chain reaction (RT-qPCR), western blot, and immunohistochemical analysis of lumbar spinal cord dorsal horn and nociceptive behaviors (thermal hyperalgesia and mechanical allodynia) measurements.

**Results:**

Herein, RT-qPCR analysis revealed that spinal *hdac3*, *hdac4*, and *hdac6* were upregulated in CCI rats. Western blotting and immunofluorescence staining further confirmed that HDAC3, HDAC4, and HDAC6 were significantly upregulated, whereas GABA and its synthesis key enzyme glutamic acid decarboxylase (GAD) 65 were dramatically downregulated. Intrathecal panobinostat attenuated nociceptive behavior and restored the downregulated spinal GAD65 and GABA expression in CCI rats.

**Conclusions:**

HDAC upregulation might induce nociception through GAD65 and GABA inhibition in CCI-induced neuropathy. These findings strongly suggest that HDACs negatively regulate inhibitory neurotransmitters, constituting a potential therapeutic strategy for an epigenetic approach to manage NP.

## Abstract 2

Supplemental Digital Content is Available in the Text.

This article presents HDAC3, HDAC4, and HDAC6 could regulate gamma-aminobutyric acid system. These findings provide a novel therapeutic approach to the management of neuropathic pain.

## 1. Introduction

Neuropathic pain (NP) is a debilitating pain syndrome caused by central or peripheral nervous system lesions with spontaneous pain, allodynia (pain evoked by nonpainful stimuli), and hyperalgesia (increased response to painful stimuli).^15^ NP affects ∼7% of the global population, and this number is increasing.^43,47^ However, studies on NP mechanisms have not been exhaustive, and NP can increase tolerance to most analgesics.^5^ Only a few treatments effectively relieve NP symptoms.^15^ Therefore, novel treatment modalities for NP management are needed.

Recently, modulation of epigenetic mechanisms, such as DNA methylation/demethylation, histone modifications, and gene expression alteration (without changing the DNA sequence) using noncoding RNAs, has emerged as a promising therapeutic strategy for NP.^12,35^ Histone deacetylases (HDACs) suppress gene transcription and may be an alternative therapeutic approach,^8,54^ including for pain.^38,40^ At least 18 mammalian HDACs have been identified and are divided into 4 classes.^32^ However, which spinal HDAC isoforms are involved in peripheral neuropathy–induced nociceptive sensitization remains unclear. This study evaluated the role of spinal zinc-dependent HDAC1–11 and acetylation at lysine 9 of histone 3 (H3K9ac) in chronic constriction injury (CCI)-induced NP. Based on quantitative real-time polymerase chain reaction (RT-qPCR) results, we also investigated HDAC3, HDAC4, and HDAC6 isoform expression in neurons, microglia, and astrocytes in the lumbar spinal cord dorsal horn (SCDH) in CCI rats.

Gamma-aminobutyric acid (GABA) is an inhibitory nociceptive neurotransmitter synthesized from glutamate by specific glutamic acid decarboxylases (GADs) in GABAergic neurons of the SCDH. Glutamic acid decarboxylases have 2 distinct isoforms: 65 kDa GAD (GAD65) and 67 kDa GAD (GAD67). GAD65 is mainly expressed in the superficial laminae of the SCDH,^31^ where nociception is integrated and controlled HDAC activation attenuates *Gad2* expression, decreasing GAD65 and GABA synthesis,^2,14^ thereby playing a vital role in the development and maintenance of NP.^55^

An HDAC inhibitor (HDACi) relieved pain by attenuating histone acetylation in rheumatoid arthritis.^9^ Furthermore, systemic or intrathecal HDACi administration ameliorated inflammatory pain.^3,7^ Several neuropathic animal studies have found that HDACi has analgesic effects.^11,24^ Panobinostat, a nonselective HDACi, is an FDA- and EMA-approved drug for cancer treatment.^25^ We previously reported that intra-articular administration of panobinostat attenuated nociception by inhibiting osteoarthritis (OA) progression in rats.^50^ However, the effects of panobinostat on nociception and NP in the central nervous system (CNS) remain unclear. In this study, we investigated whether the analgesic mechanism of panobinostat involves the regulation of HDAC-mediated GAD65 and GABA expression in NP.

## 2. Method and material

### 2.1. Animals

Adult male Wistar rats (BioLASCO Taiwan Co, Ltd, Taipei, Taiwan) weighing 250 to 285 g were used in this study. All rats were anesthetized with 2%–3% isoflurane inhalation for surgery. All experiments were approved by the National Sun Yat-sen University Institutional Committee for the Care and Use of Animals (Approval No. IACUC-10643) and abided by the Guide for the Care and Use of Laboratory Animals published by the National Research Council and ARRIVE guidelines. After the nociceptive behavioral experiment, the rats were euthanized for spinal cord examination. Only rats without hematomas or spinal cord injuries were chosen for behavioral analysis.

### 2.2. Peripheral neuropathy induction and intrathecal catheter placement

After catheterization, the rats underwent CCI surgery on the right sciatic nerve (middle part of the thigh) as described previously.^4^ In the sham group, the right sciatic nerve was surgically exposed without ligation. The intrathecal catheter was implanted as described previously.^53^ For the prophylactic analgesic effect of the panobinostat experiment, rats were randomly divided into 3 groups: sham group, CCI groups, and CCI + panobinostat group (daily intrathecal panobinostat [1 µg/d] administration for 27 days after CCI). Panobinostat was administered in 10-µL artificial cerebrospinal fluid comprising 2.6 mM K^+^, 21.0 mM HCO_3_^−^, 151.1 mM Na^+^, 1.3 mM Ca^2+^, 3.5 mM glucose, 0.9 mM Mg^2+^, 2, 5 mM HPO_4_^2−^, and 122.7 mM Cl^−^.

### 2.3. Thermal hyperalgesia assessment

The rats were placed in compartments of clear plastic cages with raised glass platforms. Thermal hyperalgesia was then tested in an IITC analgesic system (IITC Inc., Woodland Hills, CA) as described previously.^44^ Licking or quick withdrawal of the leg was considered a positive pain signal. The threshold of paw withdrawal latency (PWL) was set at 30 seconds, and the mean PWL was averaged across 3 positive tests.

### 2.4. Mechanical allodynia assessment

After the rats were settled in clear plastic cage compartments with raised metal mesh floors to allow easy access to the rat's hind paw, their mechanical allodynia was measured using von Frey filaments (Stoelting, Wood Dale, IL) as previously described.^44^ Licking or quick withdrawal of the leg was considered a positive pain signal. The maximum paw withdrawal threshold (PWT) weight, determined using Chaplan “up–down” method, was limited to 10 g, and the mean PWT was averaged from 3 positive tests.

### 2.5. Quantitative real-time polymerase chain reaction

After CCI, the rats were sacrificed on days 3, 7, 14, and 28, and enlarged SCDHs were collected by exsanguination. RNA extraction and RT-qPCR were performed as previously described.^52^ cDNA products were subjected to RT-qPCR using 2X SYBR Green PCR master mix (Roche Diagnostics, Indianapolis, IN) in a StepOnePlus Real-Time PCR system and expressed as fold changes over that in the control experiments. The primer sequences are shown in Table 1.

**Table 1:** Quantitative real-time polymerase chain reaction primer sequences.

Gene	Gene no.	Forward primers	Reverse primers
hdac1	NM_001025409.1	CTC​ACC​GAA​TCC​GAA​TGA​CT	AGC​CAT​CAA​ATA​CCG​GAC​AG
hdac2	NM_053447.1	TGC​TGT​CCT​CGA​GCT​ACT​GA	TCC​CTC​ATG​GGA​AAG​TTG​AC
hdac3	NM_053448.1	CTA​GAC​CAG​ATC​CGC​CAG​AC	TGG​CCT​GCT​GTA​GTT​CTC​CT
hdac4	NM_053449.1	ACG​GTC​AAG​GCT​TAA​GCA​GA	ACG​TTG​CCA​GAG​CTG​CTA​TT
hdac5	NM_053450.1	ATC​GCT​ACG​ACA​ATG​GGA​AC	CCA​GCG​GAG​ACT​AGA​ACG​AC
hdac6	XM_006256755.2	TCA​GCG​CAG​TCT​TAT​GGA​TG	GCG​GTG​GAT​GGA​GAA​ATA​GA
hdac7	XM_006226246.3	TGG​AGA​CAA​CAG​CAA​GCA​TC	TCC​CGT​TAT​CCA​GTT​TGA​GG
hdac8	NM_001126373.2	TTT​TCC​CAG​GAA​CAG​GTG​AC	CTG​CTC​CCA​GCT​GTA​GAA​CC
hdac9	NM_001200045.1	CTC​AGA​GCC​CAA​CTT​GAA​GG	GAA​CTG​AAG​CCT​CGT​TTT​CG
hdac10	NM_001035000.1	CCT​GGA​TGG​GCA​GAT​AAG​AA	AAC​CCT​CCA​GTT​GTC​TGT​GG
hdac11	NM_001106610.2	CAC​CTT​CAT​GGG​CCT​AGA​GA	TTG​AGA​TAG​CGC​CTT​GTG​TG

### 2.6. Western blot analysis

Western blot analysis was performed as previously described.^45,51^ Briefly, polyvinylidene difluoride membranes were incubated with primary antibodies (Table 2). Subsequently, the membranes were incubated with horseradish peroxidase–conjugated secondary antibodies and examined by chemiluminescence. We used β-actin as the internal control for protein loading. Band density against the background was measured using densitometry. We used a UVP BioChemi imaging system (UVP LCC, Upland, CA) to acquire images and LabWorks 4.0 software (UVP LCC) to quantify relative density.

**Table 2:** Primary antibodies for western blot and immunofluorescence staining.

	Antigen	Host	Catalog	Supplier	Dilution ratio
Western blot	Anti-HDAC3	Rabbit	ab32369	Abcam, Cambridge, MA	1:10000
	Anti-HDAC4	Rabbit	ab12172	Abcam	1:1000
	Anti-HDAC6	Rabbit	GTX100722	GeneTex, Irvine, CA	1:5000
	Antihistone H3 (acetyl K9; H3K9)	Rabbit	ab4441	Abcam	1:500
	Anti-β-actin	Mouse	A5441	Sigma-Aldrich, St Louis, MO	1:5000
Immunofluorescence staining	Antineuronal nuclei Alexa Fluor 488 (NeuN)	Mouse	MAB377X	EMD Millipore, Temecula, CA	1:1000
	Antiglial fibrillary acidic protein (GFAP)	Mouse	MAB3492	EMD Millipore	1:1000
	Anti-CD11b (OX42)	Mouse	CBL1512	EMD Millipore	1:500
	Antihistone H3 (acetyl K9; H3K9)	Rabbit	ab4441	Abcam	1:500
	Anti-HDAC3	Rabbit	ab32369	Abcam	1:500
	Anti-HDAC4	Rabbit	ab12172	Abcam	1:500
	Anti-HDAC6	Rabbit	GTX100722	GeneTex,	1:100
	Anti-GAC65	Rabbit	ab26113	Abcam	1:100
	Anti-GABA	Rabbit	GTX125988	GeneTex	1:500

GABA, gamma-aminobutyric acid; HDAC, histone deacetylase.

### 2.7. Histone deacetylase activity assay

The HDAC in harvested spinal cord samples was detected using an HDAC activity fluorometric assay kit (Catalog #K330-100, BioVision, Inc., Milpitas, CA). To a 96-well plate, standard buffer (0, 2, 4, 6, 8, and 10 μM; 42.5 μL/well), 10X HDAC assay buffer (5 μL/well), and HDAC fluorometric substrate (2.5 μL/well) were added to a final mixing volume of 50 μL, followed by incubation at 37°C for 30 minutes. Then, 5 μL/well of lysine developer was added to stop the reaction. After incubating at 37°C for 30 minutes, an Ex/Em wavelength of 350 to 380/440 to 460 nm was used to measure the fluorescent signal.

### 2.8. Immunofluorescence assay

Immunohistochemistry and image quantification were performed as described.^19^ SCDH sections were incubated with primary antibodies (Table 2). Then, the slices were incubated with Alexa Fluor 488 or Cy 3-conjugated secondary antibodies. Leica DM-6000B fluorescence microscope (Leica, Wetzlar, Germany) equipped with an integrated SPOT Xplorer digital camera (Diagnostic Instruments Inc., Sterling Heights, MI) was used to examine the sections. SPOT software 4.6 (Diagnostic Instruments Inc) was used for analysis. ImageJ software 2.9.0 (National Institutes of Health, Bethesda, MD) was used to quantify the immunofluorescent images from laminae I−III in the SCDH. The average of 3 rats per group was calculated. Acquisition parameters and image size remained the same for all conditions of the SCDH sections on the same slide.

### 2.9. Statistical analysis

Data are presented as mean ± standard error of the mean. Groups were compared using one-way analysis of variance followed by Tukey post hoc tests. A *P*-value of <0.05 was considered statistically significant, and analysis was performed using SigmaPlot version 12.0 (Systat Software, Inc., San Jose, CA).

## 3. Results

### 3.1. Chronic constriction injury–induced histone deacetylase mRNA expression in the dorsal lumbar spinal cord

**Figure 1.**
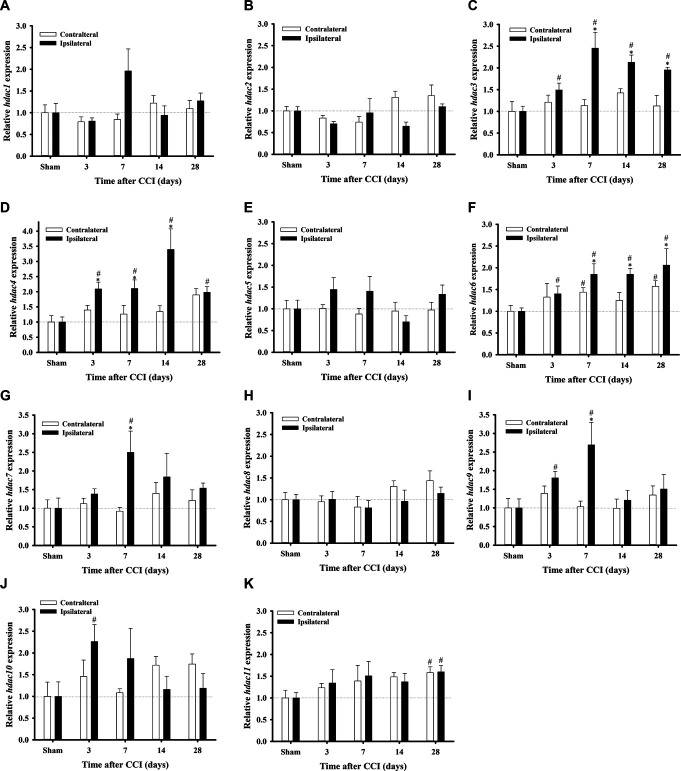
Time-course changes in *hdac1–11* mRNA expression after CCI of the rat spinal cord. *hdac1–11* expression in the ipsilateral and contralateral lumbar SCDH in sham and CCI rats on days 3, 7, 14, and 28 after surgery evaluated by RT-qPCR. *hdac1* (A), *hdac2* (B), *hdac3* (C), *hdac4* (D), *hdac5* (E), *hdac6* (F), *hdac7* (G), *hdac8* (H), *hdac9* (I), *hdac10* (J), and *hdac11* (K) mRNA levels in the ipsilateral and contralateral SCDH after CCI surgery (n = 5 at each group and each time point). *P < 0.05 compared with the contralateral side of each group. #*P* < 0.05 compared with the same side of the sham group. CCI, chronic constriction injury; RT-qPCR, quantitative real-time polymerase chain reaction; SCDH, spinal cord dorsal horn.

Through RT-qPCR, *hdac1–11* expression in the lumbar SCDH of sham-operated and CCI rats was examined on days 3, 7, 14, and 28 after surgery. Seven days after CCI, ipsilateral *hdac1* expression showed a slight increase compared with the sham group (*P* = 0.056) and the contralateral side (*P* = 0.061; Fig. 1A). However, CCI did not cause any notable difference on *hdac2* in SCDH (Fig. 1B). *hdac3* expression in the ipsilateral side was upregulated significantly on days 3 (*P* = 0.016), 7 (*P* < 0.001), 14 (*P* = 0.005), and 28 (*P* = 0.02) after CCI compared with the sham group. In addition, it showed significant differences on days 7, 14, and 28 after CCI compared with the contralateral side (Fig. 1C). *hdac4* expression in the ipsilateral side increased significantly on days 3 (*P* = 0.008), 7 (*P* = 0.016), 14 (*P* = 0.0035), and 28 (*P* = 0.004) after CCI compared with the sham group. Furthermore, *hdac4* expression in the ipsilateral side showed significant differences compared with the contralateral side on days 3 (*P* = 0.018), 7 (*P* = 0.031), and 14 (*P* < 0.001) after CCI (Fig. 1D). *hdac6* expression increased significantly on days 3 (*P* = 0.031), 7 (*P* = 0.004), 14 (*P* < 0.001), and 28 (*P* = 0.023) after CCI compared with the sham group. *hdac6* expression in the ipsilateral side also showed a significant difference compared with the contralateral side on days 7 (*P* = 0.049), 14 (*P* = 0.013), and 28 (*P* = 0.045) after CCI. *hdac6* expression in the contralateral side was also upregulated significantly on days 7 (*P* = 0.014) and 28 (*P* = 0.006) after CCI compared with the sham group (Fig. 1F). *hdac7* expression in the ipsilateral side only increased significantly on day 7 (*P* = 0.02) after CCI compared with the sham group and the contralateral side (Fig. 1G). *hdac9* expression in the ipsilateral side was upregulated significantly on days 3 (*P* = 0.011) and 7 (*P* = 0.013) after CCI compared with the sham group. Seven days after CCI, *hdac9* expression in the ipsilateral side was upregulated significantly on day 7 day (*P* = 0.031) after CCI compared with the contralateral side (Fig. 1I). *hdac10* expression in the ipsilateral side increased significantly on day 3 (*P* = 0.02) after CCI compared with the sham group but showed no significant change compared with the contralateral side (Fig. 1J). *hdac11* expression in the contralateral and ipsilateral sides increased significantly only on day 28 (*P* = 0.016 and 0.01, respectively) after CCI compared with the sham group (Fig. 1K). *hdac2*, *hdac5*, and *hdac8* expressions in the ipsilateral side did not increase significantly compared with the sham group and the contralateral side from days 3 to 28 after CCI (Fig. 1B, E, H). Based on the above results, *hdac3*, *hdac4*, and *hdac6* on the ipsilateral side were upregulated significantly from days 3 to 28 after CCI and showed significant differences from the contralateral side. Overall, the expression of *hdac1*, *hdac2*, *hdac5*, and *hdac7–11* did not present persistent changes after CCI. These findings indicate that *hdac3*, *hdac4*, and *hdac6* might play vital roles in NP and are worthy of further investigation.

### 3.2. Chronic constriction injury–induced upregulation of HDAC3, HDAC4, and HDAC6 protein expression in the spinal cord

**Figure 2.**
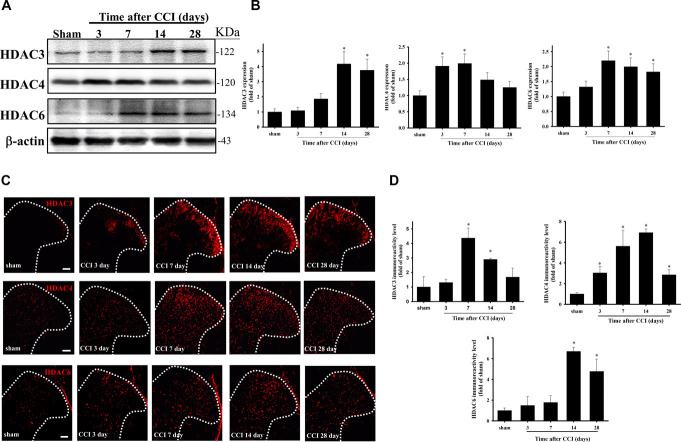
Upregulation of spinal HDAC3, HDAC4, and HDAC6 protein expressions in CCI rats. (A) Western blot analysis of HDAC3, HDAC4, and HDAC6 expression in the ipsilateral SCDH on days 3, 7, 14, and 28 after CCI. β-actin was used as the internal control. (B) HDAC3, HDAC4, and HDAC6 expression levels were quantified and normalized to β-actin level. (C) Topographic upregulation of HDAC3, HDAC4, and HDAC6 expression in the superficial gray matter of the lumbar SCDH after CCI (red). (D) Quantification of immunofluorescence reactivity of the time-course of HDAC3, HDAC4, and HDAC6 immunoreactivity within laminae I–III of the SCDH. CCI can upregulate HDAC3, HDAC4, and HDAC6 expression in the ipsilateral lumbar SCDH, especially in laminae I—III. *P < 0.05 compared with the sham group. Scale bar: 100 µm (n = 5 at each time point were used). CCI, chronic constriction injury; HDAC, histone deacetylase; SCDH, spinal cord dorsal horn.

To verify whether CCI affected HDAC3, HDAC4, and HDAC6 protein upregulation, we performed the western blot (Fig. 2A, B, and Supplemental Figure S1, http://links.lww.com/PR9/A261) and immunofluorescence (Fig. 2C, D) of the ipsilateral lumbar SCDH. Compared with the sham group through western blot, CCI increased HDAC3 expression significantly on days 14 (*P* = 0.006) and 28 (*P* = 0.017) after CCI. HDAC4 expression was markedly upregulated on days 3 (*P* = 0.015) and 7 (*P* = 0.012) after CCI. HDAC6 expression was upregulated significantly on days 7 (*P* = 0.032), 14 (*P* = 0.024), and 28 (*P* = 0.018) after CCI (Fig. 2A, B, and Supplemental Figure S1, http://links.lww.com/PR9/A261). Compared with the sham group by immunofluorescence (red), HDAC3 immunoreactivity increased significantly on days 7 (*P* = 0.014) and 14 after CCI (*P* = 0.049), and HDAC4 immunoreactivity was rapidly and persistently upregulated by CCI on the ipsilateral side, starting at day 3 (*P* = 0.0323), peaking at day 14 (*P* = 0.009), and maintained until day 28 after CCI (*P* = 0.022). In addition, compared with the sham group and the contralateral side, HDAC6 immunoreactivity increased significantly on days 14 (*P* = 0.001) and 28 (*P* = 0.014) after CCI (Fig. 2C, D). According to western blot and immunofluorescence results, CCI-induced nociception upregulated HDAC3, HDAC4, and HDAC6 protein expression in laminae I—III of the rat ipsilateral lumbar SCDH.

### 3.3. Chronic constriction injury attenuated spinal H3K9 acetylation expression in the spinal cord

**Figure 3.**
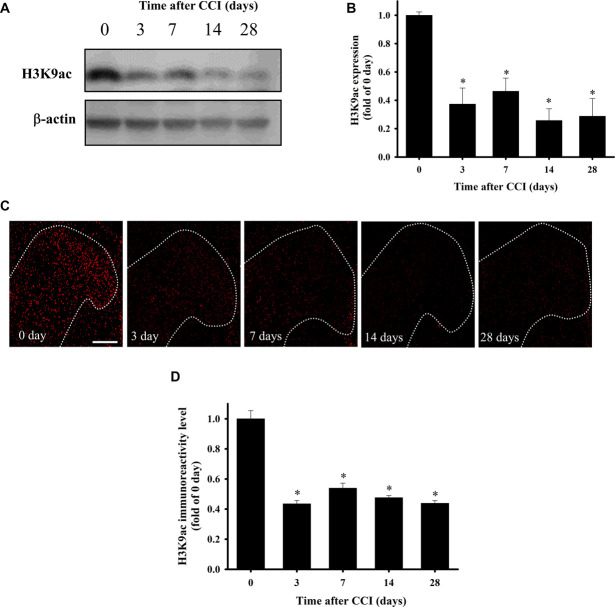
Effect of CCI on H3K9ac expression in the lumbar spinal cord. (A) Western blot of H3K9ac expression in the ipsilateral lumbar SCDH from sham and 7, 14, and 28 days after CCI. (B) Quantification of western blot results of H3K9ac protein expression normalized to β-actin. (C) Representative H3K9ac immunofluorescence (red) in laminae I–III of the ipsilateral lumbar SCDH of sham and CCI rats at postoperative days 3, 7, 14, and 28. (D) Quantification result of immunofluorescence reactivity of H3K9ac in the ipsilateral lumbar SCDH compared with 0 days. CCI-induced neuropathy can downregulate H3K9ac in the spinal cord. *P < 0.05 compared to the 0 day. Scale bar: 200 µm (n = 5 at each time point were used). CCI, chronic constriction injury; SCDH, spinal cord dorsal horn.

H3K9 acetylation (H3K9ac) is dominant in histone deposition and chromatin assembly. We assessed whether CCI-induced HDAC upregulation attenuated H3K9ac expression. Figure 3 shows western blotting (Fig. 3A, B, and Supplemental Figure S1, http://links.lww.com/PR9/A261) and immunofluorescence staining (Fig. 3C, D) of H3K9ac in the ipsilateral lumbar SCDH on days 3, 7, 14, and 28 after CCI. H3K9ac protein levels were quantified and normalized against β-actin level and expressed vs the sham group, respectively (Fig. 3B, D). H3K9ac expression decreased significantly from days 3 to 28 (*P* < 0.001) after CCI compared with the sham group (Fig. 3B) by western blotting quantification. Similarly, immunofluorescence staining revealed significant CCI-induced downregulation of H3K9ac in laminae I–III of the SCDH (*P* < 0.001) (Fig. 3D). Hence, nociception downregulates H3K9ac expression in SCDH.

### 3.4. Chronic constriction injury upregulates HDAC3, HDAC4, and HDAC6 expression in neuronal cells

**Figure 4.**
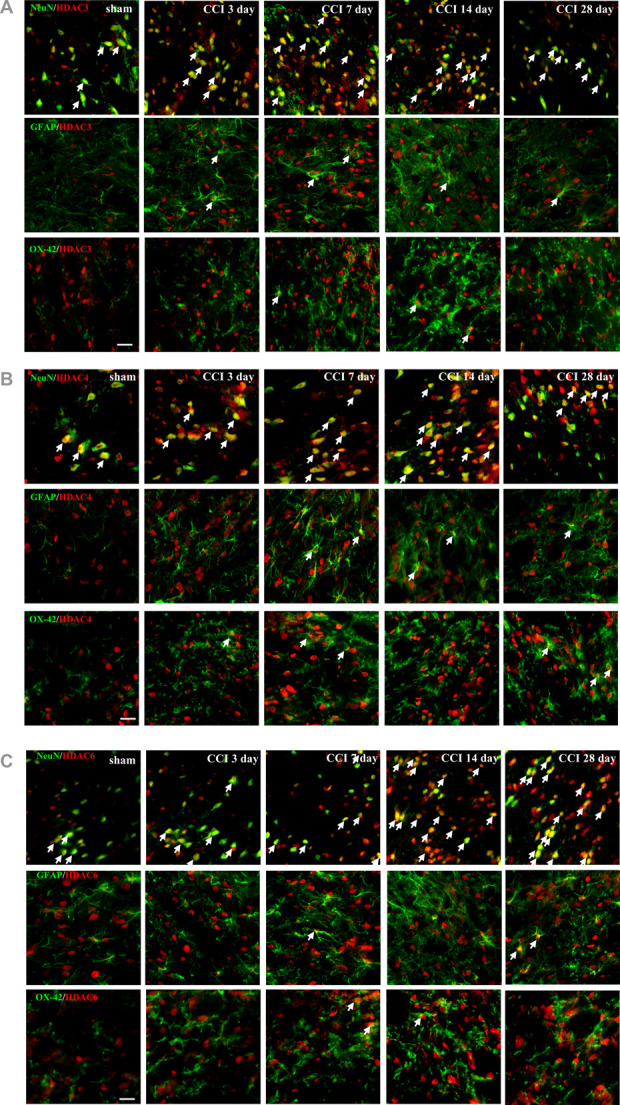
Immunohistochemical colocalization of HDAC3, HDAC4, and HDAC6 with neurons, astrocytes, and microglia in the ipsilateral superficial lumbar SCDH after CCI. Representative HDAC3 (A), HDAC4 (B), and HDAC6 (C) red immunofluorescence colocalized with NeuN (a neuronal-specific marker), GFAP (an astrocyte-specific marker), and OX-42 (a microglial cell–specific marker) green expression in sham and 3, 7, 14, and 28 days after CCI. The merged images (yellow) indicate colocalization of the HDACs with NeuN- and fewer GFAP-immunoreactive cells. Scale bar: 25 µm. CCI, chronic constriction injury; GFAP, glial fibrillary acidic protein; HDAC, histone deacetylase; SCDH, spinal cord dorsal horn.

We performed double immunofluorescence staining of HDAC3, HDAC4, and HDAC6 (red) with 3 specific cell markers (green), NeuN (a neuronal-specific marker), glial fibrillary acidic protein (an astrocyte-specific marker), and OX-42 (a microglial cell-specific marker) in ipsilateral lumbar SCDH after CCI (Fig. 4A–C). HDAC3, HDAC4, and HDAC6 colocalized with neurons and were less visible in astrocytes and microglia from day 3 to 28 after CCI and in the sham group. Chronic constriction injury–induced HDAC3, HDAC4, and HDAC6 colocalized with neurons and exhibited relatively higher expression on day 14 after CCI (yellow).

### 3.5. Analgesic effect of the histone deacetylase inhibitor panobinostat in chronic constriction injury rats

**Figure 5.**
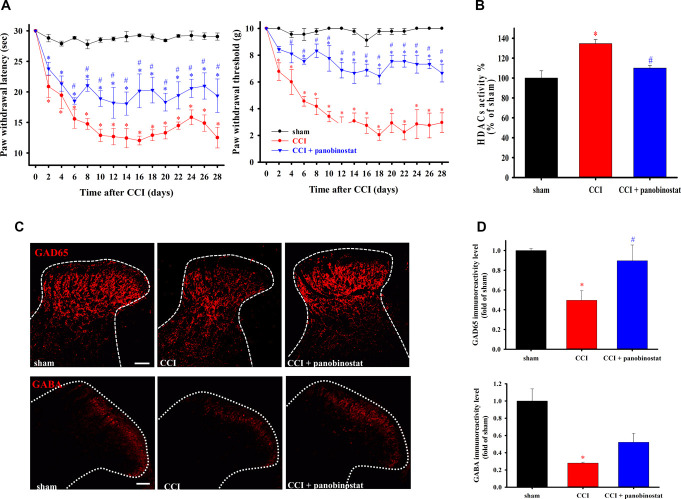
The inhibited effect of panobinostat on CCI-induced nociception, HDACs activity, and GAD65 and GABA downregulation. (A) Time-course for thermal hyperalgesia and mechanical allodynia in CCI rats receiving daily intrathecal panobinostat (1 µg/d) from day 1 to 27 after surgery. (B) The total HDACs activity of the ipsilateral lumbar SCDH tissue of sham group, day 28 after CCI, and day 28 after CCI with daily intrathecal panobinostat injection were assayed using the fluorometric HDAC activity assay kit. (C) Immunofluorescence staining images show GAD65 and GABA in laminae I–III of the SCDH from the sham, the CCI, and the CCI + panobinostat groups. (D) Quantification of GAD65 and GABA immunoreactivity. Panobinostat administration significantly attenuates CCI-induced nociceptive, HDACs activity, and downregulation of GAD65 and GABA immunoreactivity in the ipsilateral SCDH. Data are presented as the mean ± standard error. * and # represent *P* < 0.05 compared with the sham and the CCI groups, respectively. Scale bar: 100 µm. CCI, chronic constriction injury; HDAC, histone deacetylase; SCDH, spinal cord dorsal horn.

As shown in Figure 5A, rats were randomly separated into 3 groups: sham, CCI, and CCI + panobinostat. Nociceptive behaviors, thermal hyperalgesia, and mechanical allodynia were assessed every 2 days after CCI. Thermal hyperalgesia and mechanical allodynia were significantly induced in rats for 28 days after CCI surgery. Compared with the CCI group, daily intrathecal administration of 1 µg panobinostat, an HDACi, significantly improved CCI-induced thermal hyperalgesia from day 7 to 28 and mechanical allodynia from day 5 to 28. In Figure 5B, tissue lysates from the ipsilateral lumbar SCDH 28 days after CCI showed that HDAC activity was significantly higher than that in the sham group (*P* = 0.014). We used panobinostat to verify whether HDACi downregulates HDAC under neuropathic conditions, and HDAC activity in the SCDH of the CCI + panobinostat group was significantly attenuated compared with the CCI group (*P* = 0.0104). GAD65, regulated by HDAC, participates in the conversion of glutamate into GABA. In Figure 5C, we performed immunofluorescence staining to verify whether GAD65 (red) and GABA (red) expression are regulated by HDACs in laminae I—III of the SCDH. The quantification results indicated that GAD65 and GABA expression was significantly attenuated in the CCI group (*P* = 0.038 and 0.03 respectively) and enhanced in the CCI + panobinostat group (*P* = 0.049 and 0.0403, respectively) (Fig. 5D). Herein, panobinostat can not only attenuate CCI-induced nociceptive sensitization but also downregulate HDAC activity, which leads to restore GAD65 expression, inhibited by HDAC, in the SCDH under neuropathy.

## 4. Discussion

Herein, we examined the mRNA expression of HDACs in the SCDH of CCI rats and found that *hdac3*, *hdac4*, and *hdac6* may play a vital role in NP (Fig. 1). We also observed that CCI significantly upregulates HDAC3, HDAC4, and HDAC6 expression (Fig. 2) but downregulates H3K9ac expression in the SCDH (Fig. 3). In addition, HDAC3, HDAC4, and HDAC6 are mainly expressed in neurons of the ipsilateral SCDH (Fig. 4). To investigate the inhibitory effects of HDACi on CCI-induced nociceptive sensitization, rats were administered panobinostat, a nonspecific HDACi. Intrathecal panobinostat inhibited CCI-induced nociceptive sensitization and spinal HDAC activation. Moreover, panobinostat restored the CCI-induced downregulation of spinal inhibitory transmitter systems GAD65 and GABA (Fig. 5).

Epigenetic mechanisms in the CNS affect gene expression without altering the underlying DNA sequence and are related to learning, memory, and synaptic plasticity.^16^ There are 3 major categories of epigenetic modification: DNA methylation, nucleosome positioning, and histone modification.^36^ Histone modification contributes to gene expression regulation, DNA repair, and DNA replication. Histone acetylation can occur at H3K9 and is removed by HDACs,^6^ which plays a vital role in transcriptional regulation.^40^ Inhibition of spinal HDAC leads to H3K9ac upregulation and suppresses nociceptive sensitization.^35^ HDACs are divided into zinc-dependent and NAD^+^-dependent families. Among 18 isoforms, most HDACs localize in the nucleus, except HDAC6, which localizes in the cytoplasm.^1^ Previous studies found that HDAC1, HDAC2, and HDAC4 are significantly expressed in the spinal cord and dorsal root ganglion of CCI Sprague–Dawley (SD) rats.^27,33,49^ Herein, we found that CCI induced HDAC3, HDAC4, and HDAC6 expression in the lumbar SCDH of Wistar rats. This is supported by previous reports indicating that inhibition of HDAC3, HDAC4, and HDAC6 attenuated nociception in different NP models, such as spinal nerve ligation (SNL) and cisplatin-induced nociceptive sensitization in SD rats.^13,17,23,28^ The differences in HDAC expression between the previous and present results are likely due to differences in animal model strains and sampling locations of the spinal cord. The lumbar SCDH processes nociceptive signals from the lower body and becomes hyperactive or sensitized during CCI-induced NP. Analysis of the SCDH may rule out non-nociceptive transmission of the spinal cord in our study.

Studies have indicated that HDAC3, HDAC4, and HDAC6 participate in neuropathic disease. Nuclear HDAC3 contributes to some neurodegenerative diseases.^48^ In SNL-induced NP animal models, HDAC3 is predominantly expressed in the neurons and microglia after surgery.^17^ HDAC3 elicits the inflammatory factors, IL-6 and TNF-α, and induces inflammatory pain by regulating the STAT and NF-κB pathways.^17,26^ HDAC4 is an effective therapeutic target for inflammatory hyperalgesia.^3^ HDAC4 is involved in sensory neuron sensitization, and downregulation of HDAC4 activity by sensory neuron-specific conditional knockout of HDAC4 leads to attenuation of thermal hypersensitivity in complete Freund adjuvant (CFA)-induced mice.^10^ Reduction of nuclear HDAC4 content without change in total HDAC4 levels combined with an elevated level of neuronal nuclear H3K9ac occurs in mouse SCDH neurons after paw inflammation in a rat model of CFA-induced inflammatory pain but not in models of capsaicin-induced inflammatory pain and spared nerve injury–induced NP. Modulation of HDAC4 activity via elevated expression of constitutively nuclear localized dominant-active HDAC4 mutant effectively inhibits both CFA-induced H3K9ac in mouse SCDH neurons and mechanical hyperalgesia/allodynia in mice, and it only reduces intraplantar formalin-induced second-phase nociceptive behaviors. However, it does not affect capsaicin-induced acute nociceptive behaviors in mice.^29^ Although CCI enhances the total expression of HDAC3/4 in the SCDH, we did not examine the cytoplasmic/nuclear ratio of HDAC3/4, and the precise mechanisms of this process remain unclear. Further study of HDAC3/4 in SCDH neurons in response to peripheral nerve injury may aid in developing new therapeutic strategies for treating NP. HDAC6 is expressed in the neuronal cytoplasm^41^ and participates in neurodegenerative diseases by mediating oxidative stress, tau protein expression, and synaptic function.^30^ Furthermore, HDAC6 regulates the intracellular transportation of mitochondria and decreases axon growth,^20^ affecting neuronal survival, memory, and disease progression.^30^ Moreover, neurodegenerative insults cause HDAC6 to translocate into the nucleus, repressing neuronal-related survival gene transcription.^30^ HDAC6 inhibitors alleviate mechanical allodynia in the mouse model of spared nerve injury–induced NP and CFA-induced inflammatory pain.^39^ Few previous reports indicate that CCI upregulates neuronal HDAC3, HDAC4, and HDAC6. Herein, we report that CCI induces neuronal HDAC3, HDAC4, and HDAC6 expression in the SCDH and participates in nociceptive sensitization.

Gamma-aminobutyric acid is a major inhibitory neurotransmitter in the spinal cord. Gamma-aminobutyric acid decreases synaptic transmission in the spinal cord resulting from glutamate-mediated neuron excitation in neuropathy.^18^ Previous studies have demonstrated that downregulating GABA and GAD expression leads to nociceptive sensitization.^46,55^ Kami et al. indicated that neuropathy attenuates GAD and GABA expression in the SCDH of partial sciatic ligation–induced neuropathic mice.^21^ There are 2 distinct GADs, GAD65 and GAD67, encoded by *Gad2* and *Gad1*, respectively.^14^ Although both GAD65 and GAD67 catalyze the conversion of glutamate into GABA, they are expressed in different lamina regions of the spinal cord. GAD65 is mainly expressed in the superficial lamina, whereas GAD67 is primarily found in the deeper lamina.^31^ Laminae I–III of the SCDH participate in neuropathy-induced nociceptive sensitization.^22^ Hence, we focused on GAD65 rather than GAD67 because the superficial lamina of the SCDH contributes to nociceptive sensitization. Previous studies demonstrated that persistent pain or NP could attenuate *Gad2* transcription and downregulate GAD65 expression through HDACs.^34,56^ Moreover, experiments have demonstrated that HDACi, such as suberoylanilide hydroxamic acid, valproate, trichostatin A, and butyrate, effectively attenuate NP.^38^ However, increasing GABA production and GAD expression through HDACi has not been clearly illustrated. Therefore, we utilized a nonselective HDACi, panobinostat, to verify whether the GAD65 and GABA expression mediation involves HDAC activation.

Previous studies indicated that histone hyperacetylation resulting from HDAC repression leads to neuroprotection.^42^ HDAC inhibition may be a therapeutic approach for NP management.^11,38^ Panobinostat is mainly used to treat multiple myeloma. Moreover, it is considered the most potent HDACi in clinical progression.^37^ It is an inhibitor of all types of HDACs with an IC50 in the nanomolar range.^25^ We previously reported that panobinostat could ameliorate OA-induced nociception and decrease OA progression by mediating HDAC4, HDAC6, and HDAC7 in the cartilage of anterior cruciate ligament transection rats.^50^ Recently, other than our previous study, there have been no investigations of panobinostat in pain treatment or for NP, warranting further research.

**Figure 6.**
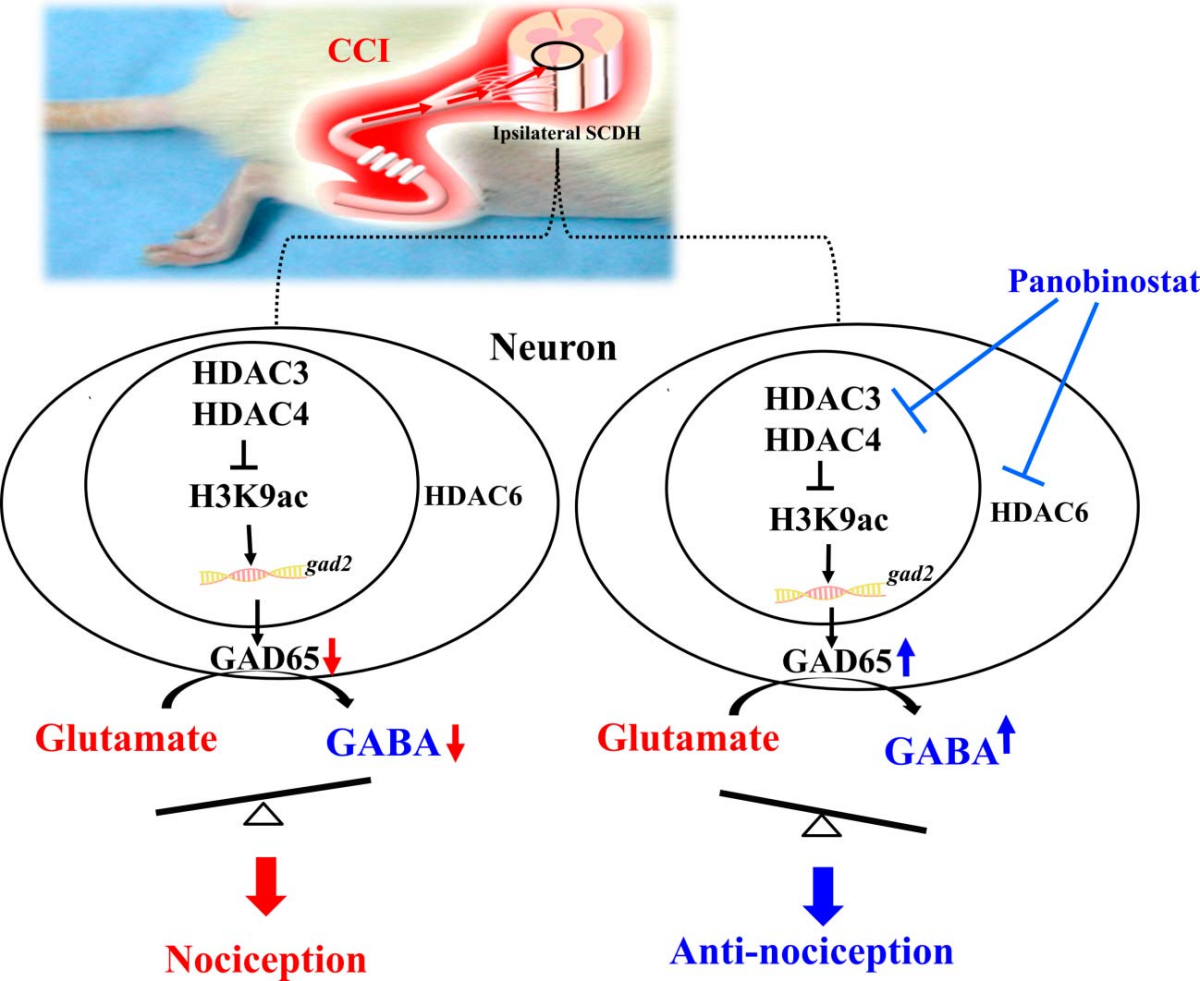
Schematic of the possible analgesic mechanism of HDACs in CCI-induced NP. CCI enhanced HDAC3, HDAC4, and HDAC6 expression in the neurons in the lumbar SCDH. Upregulating HDAC activity downregulates H3K9ac expression in the nucleus, inhibiting Gad2 expression and decreasing GAD65 expression. Downregulated GAD65 decreases the decarboxylation of glutamate and attenuates glutamate transformation into the inhibitory neurotransmitter, GABA. Thus, the excitatory glutamate and inhibitory GABA amounts are increased and reduced, respectively. Increased glutamate and decreased GABA contribute to nociceptive sensitization. Intrathecal panobinostat inhibits HDAC activity and restores the downregulation of GAD65 and GABA synthesis in the lumbar SCDH of CCI rats. Since GAD65 is restored after panobinostat treatment, GABA production may also increase, leading to pain relief. CCI, chronic constriction injury; HDAC, histone deacetylase; NP, neuropathic pain; SCDH, spinal cord dorsal horn.

The hypothetical molecular mechanisms of spinal HDAC on neuropathy-induced nociceptive sensitization are proposed in Figure 6. This study provides evidence that neurons are the primary cellular source of HDAC3, HDAC4, and HDAC6 in the ipsilateral SCDH produced in response to CCI in rats. Chronic constriction injury–induced HDAC3 and HDAC4 upregulation in neuronal nuclei can attenuate H3K9ac expression. The activation of HDACs represses *gad2* by attenuating H3K9ac expression, resulting in GAD65 downregulation, which decreases the production of GABA (inhibitory neurotransmitter) but increases that of glutamate (excitatory neurotransmitter), and both contribute to nociceptive sensitization. Pharmacological intervention confirmed that intrathecal panobinostat administration attenuated CCI-induced H3K9ac and restored GAD65 by inhibiting HDAC activity. Inhibition of HDAC3- and HDAC4-induced restoration of GAD65 could increase GABA synthesis and decrease glutamate levels. Thus, the CCI-induced upregulation of nervous cytoplasmic HDAC6 may contribute to nociceptive sensitization. Panobinostat can potentially suppress HDAC6 activity directly, resulting in analgesic effects.

## 5. Conclusions

In this article, we found that neuronal HDAC3, HDAC4, and HDAC6 expression increased significantly after CCI. After HDACi administration, HDAC activity and nociception were attenuated, accompanied by restoration of GAD65 and GABA expression in the SCDH. We conclude that HDACs mediate GABA production in NP, which may be a potential therapeutic approach. Further research is needed to elucidate the underlying cellular mechanisms.

## Disclosures

The authors have no conflict of interest to declare.

## Appendix A. Supplemental digital content

Supplemental digital content associated with this article can be found online at http://links.lww.com/PR9/A261.

## Supplementary Material

SUPPLEMENTARY MATERIAL
